# Visual detection of *Brucella* in bovine biological samples using DNA-activated gold nanoparticles

**DOI:** 10.1371/journal.pone.0180919

**Published:** 2017-07-18

**Authors:** Dheeraj Pal, Nongthombam Boby, Satish Kumar, Gurpreet Kaur, Syed Atif Ali, Julien Reboud, Sameer Shrivastava, Praveen K. Gupta, Jonathan M. Cooper, Pallab Chaudhuri

**Affiliations:** 1 Division of Bacteriology and Mycology, Indian Veterinary Research Institute, Izatnagar, India; 2 Division of Animal Biotechnology, Indian Veterinary Research Institute, Izatnagar, India; 3 Division of Biomedical Engineering, School of Engineering, University of Glasgow, Glasgow, United Kingdom; Queen's University at Kingston, CANADA

## Abstract

Brucellosis is a bacterial disease, which, although affecting cattle primarily, has been associated with human infections, making its detection an important challenge. The existing gold standard diagnosis relies on the culture of bacteria which is a lengthy and costly process, taking up to 45 days. New technologies based on molecular diagnosis have been proposed, either through dip-stick, immunological assays, which have limited specificity, or using nucleic acid tests, which enable to identify the pathogen, but are impractical for use in the field, where most of the reservoir cases are located. Here we demonstrate a new test based on hybridization assays with metal nanoparticles, which, upon detection of a specific pathogen-derived DNA sequence, yield a visual colour change. We characterise the components used in the assay with a range of analytical techniques and show sensitivities down to 1000 cfu/ml for the detection of *Brucella*. Finally, we demonstrate that the assay works in a range of bovine samples including semen, milk and urine, opening up the potential for its use in the field, in low-resource settings.

## Introduction

Brucellosis is a bacterial disease caused by *Brucella* species (facultatively intracellular *coccobacilli*) and remains the world’s most common zoonoses.[1] Ten species have so far been identified and out of these, four classical species (*Brucella melitensis*, *Brucella suis*, *Brucella abortus* and *Brucella canis*) have been reported to be responsible for human infection.[2,3] Because of its non-specific clinical features and the lack of efficient diagnostic methods, the number of undetected cases remains high.[4] Brucellosis is also linked to a number of serious challenges in the economy of developing countries, including India.[5] The control of brucellosis in humans first requires its control in animals, leading to large programmes of vaccinations. However, this strategy cannot succeed without the ability to detect the reservoir animals in a robust and efficient way, so that they can be removed from the herd. Given the local infrastructure around many farms in rural India, there is a need to be able to provide a test that is sensitive and which can be detected visually. For example, rural veterinary clinics often do not have access to essential lab equipment necessary to run current gold standard tests, while access to expertise (for the preparation of reagents, assay processing and result interpretation) can also be lacking.

Currently, the isolation of the bacteria from clinical samples and the detection of anti-*Brucella* antibodies from suspected animals are the two primary methods employed for the diagnosis of brucellosis. Culture and isolation are time-consuming and labour intensive processes: the organism takes a minimum of 4–5 days to grow and the culture should be kept at least 45 days to rule out false negatives.[6] There is also the need for culture facilities, often associated with a reference laboratory (certainly not always available in Low and Medium Income Countries, LMICs).

Alternatively, serological tests (e.g. Rose Bengal Plate Agglutination Test (RBPT), Standard Tube Agglutination Test (STAT)). are based on the antigenic properties of bacterial lipopolysaccharides (LPS).[7] These not only require blood sampling and processing but also have been shown to lead to high cross reactivity with other organisms (e.g. *Yersinia enterocolitica* O:9,*Vibrio cholerae*, *Escherichia coli* O: 157, *Francisella tularensis*, *Salmonella urbana* group N and *Stenotrophomonas maltophilia*[8]). The test therefore generally has a low specificity.

Nucleic acid tests (NATs) offer high performance by bridging the ability to identify the pathogen (similarly to culture) with the speed of molecular-based assays. As a consequence *Brucella spp* have been identified using multiplexed PCR [9] as well as isothermal LAMP assays.[10] Both methods have limited application in the field as they require well-equipped laboratory and skilled personnel, not available in many rural settings in LMICs.

In contrast, hybridization tests based on metal nanoparticles are simple to implement and can be read out visually through a colour change, arising from the plasmonic interactions between the probes,[11] making them an attractive system for diagnostic applications. Gold in particular offers the possibility of using a colour shift in the visible range, from red to purple,[12] when aggregated, usually through the addition of simple reagents such as salts, and has thus been preferred in the implementation of these tests for the molecular detection of pathogens.[13]

**Fig 1:**
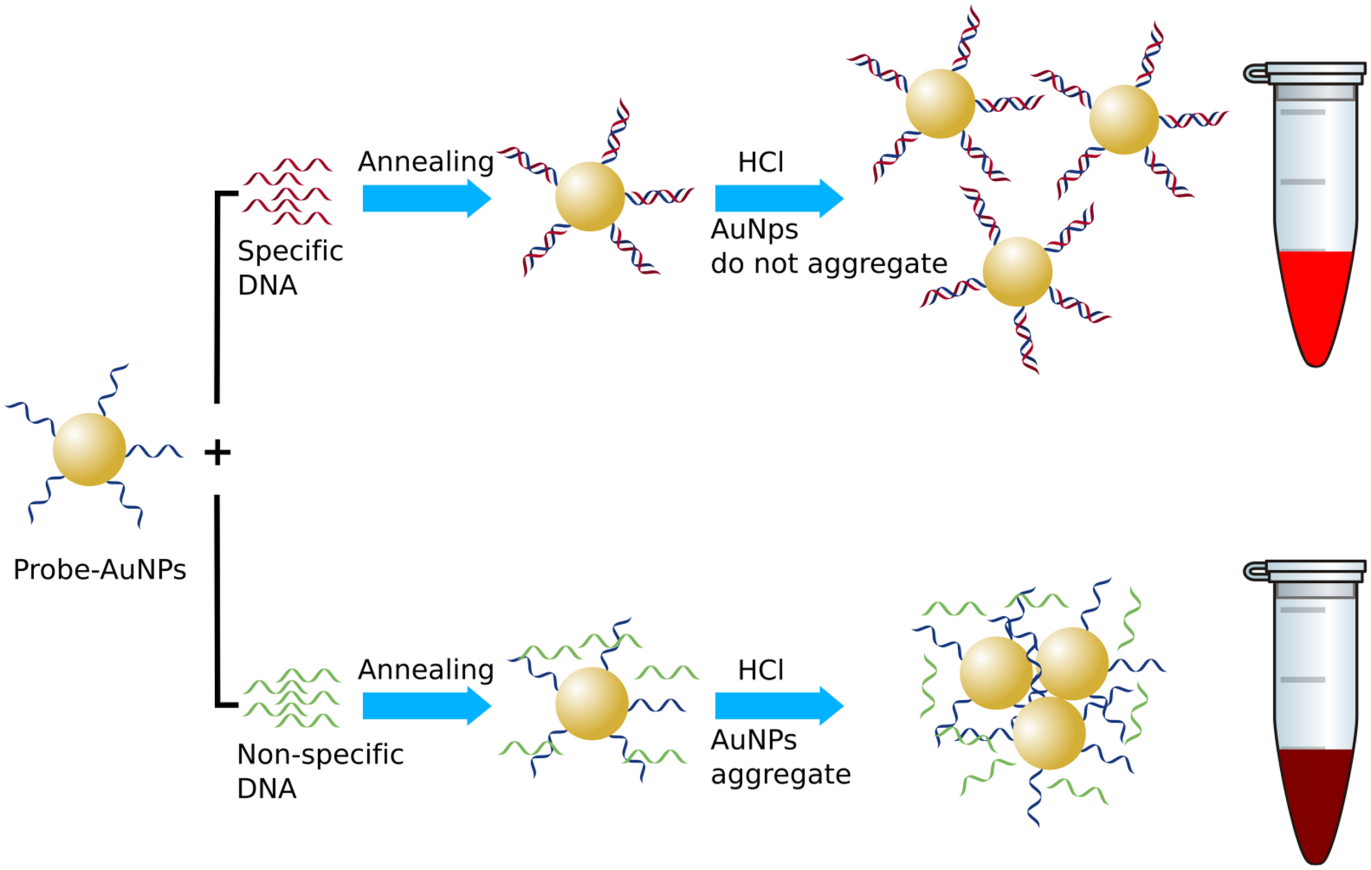
Schematic representation of the visual detection of *Brucella* based on salt induced aggregation. a) positive reaction; and b) negative reaction. After hybridisation of the AuNP-probe with the specific DNA (positive sample), the negative charge surrounding the AuNPs is increased. After adding salts (here HCl), the negative charges surrounding the unhybridised AuNP are neutralised resulting in the aggregation of AuNP (change to purple colour), whereas in the case of a positive sample (with more negative charge), the negative charges are not neutralised completely resulting in the non-aggregation of AuNP (the colour remains red).

In this study, we designed a gold nanoparticle (AuNP) based hybridization assay to detect *Brucella* (Fig 1). This broad strategy has been successfully demonstrated previously,[14] using a probe specific to IS711, an insertion sequence with multiple copies.[15] Here we designed a thiol modified probe specific to BCSP31 a genus specific gene which codes for outer membrane protein.^16^ The direct visual detection of *Brucella* was optimized and the analytical sensitivity and specificity of the test was assessed. Importantly we show that the AuNP-oligo probe can be used for the simple, visual detection of *Brucella* from a broad range of bovine samples, including semen, milk and urine.

## Materials and methods

### Reagents

Gold (III) chloride hydrate, HAuCl4 (Sigma, USA); Sodium citrate tribasic (Sigma); Tris (2-carboxyethyl)- phosphine, TCEP (Sigma); Sodium Chloride, NaCl (SRL); Sodium dodecyl sulphate, SDS (Sigma); disodium hydrogen phosphate, Na_2_HPO_4_ (Merck, Germany); sodium dihydrogen phosphate, NaH_2_PO_4_ (Merck); sodium hydroxide, NaOH (SRL, India); potassium dihydrogen phosphate, KH_2_PO_4_ (Merck). All the chemical solutions and buffers were prepared using sterile double distilled water (ddH_2_O).

Sequences for thiol-modified oligonucleotide probe and complimentary sequence were as follows and custom synthesized:

BCSP31 probe—5’SH–(CH2)9GGGCAAGGTGGAAGATTTGCGCCT-3’

BCSP31complementary—5’-AGGCGCAAATCTTCCACCTTGCCC-3’

### Bacterial strains

*Brucella abortus S19* was used as the source of *Brucella* DNA. The organism was cultured in *Brucella* broth (Difco) incubated at 37°C, 180 rpm for 3–4 days. Non-*Brucella* species were obtained from the divisional laboratory of Indian Veterinary Research Institute, UP, India. Pure bacterial cultures were enumerated by the serial dilution method.

### Preparation of AuNP

A colloidal solution of AuNP was prepared by citrate reduction of gold (III) chloride hydrate (HAuCl_4_) as described in the literature.[16] All the glassware was first washed with aqua regia (HCl:HNO_3_ = 3:1) and rinsed with double distilled water (ddH_2_O). 250 ml of aqueous solution of HAuCl_4_ (1 mM) was allowed to boil on hot plate magnetic stirrer. While boiling, 25 ml of 38.8 mM pre boiled sodium citrate tribasic was poured quickly, which led to a change in colour from pale yellow to ruby red. The solution was refluxed for another 20 min. The solution was cooled overnight at room temperature and stored at 4°C in an opaque coloured bottle until used. The AuNP preparation was characterized spectrophotometrically (at 520 nm). The size of AuNP was also determined by transmission electron microscopy (Jem-1011 electron microscope, Jeol, USA). A drop of sample solution was loaded over a carbon coated copper grid. The sample was dried at 37°C and observed at an accelerating voltage at 80 KV.

### Functionalization of AuNP

AuNPs were functionalized by conjugation with thiol modified oligonucleotide probes following previously published protocols, with minor modifications.[17] Before conjugation, 8 μl of 2.5 μmol.l^-1^ thiol modified oligo probe was first reduced with 30 μl of 0.1 M TCEP following incubation for 1 h at room temperature (RT). After incubation, 5 μl of 3 M sodium acetate (pH 5) was added.

The DNA probe was allowed to precipitate by adding 20 μl of ethanol at -20°C for 20 min. The solution was centrifuged at 12,000 rpm for 10 min. The pellet was air dried and resuspended in 100 μl of nuclease free water. 1 ml of 1 mM AuNP was added and incubated at 25°C for 1 day in a shaker incubator. Using 100 mM sodium phosphate buffer (pH 7), the final AuNP-probe solution was diluted to 9 mM phosphate buffer. SDS (final concentration of 0.1% (w/v)) was added and the solution incubated for 30 min room temperature. The volume of buffer (2 M NaCl in 10 mM phosphate buffer, pH 7) to make a final concentration of 0.3 M NaCl was added over 2 days in 6 intervals.

The AuNP-probe was allowed to equilibrate overnight at room temperature and was centrifuged at 13,000 rpm for 15 min. The soft pellet was washed with 500 μl of washing buffer (10 mM PBS, pH 7.4, 150 mM NaCl). Finally, the pellet was resuspended in 500 μl of the assay buffer (10 mM PBS, pH 7.4, 150 mM NaCl, 0.1% SDS) and stored at RT until used.

### Probe design

Probe specific to BCSP31 gene of *Brucella* was designed in Genetool using gene information available in NCBI (accession no. KF56031). Sequence homologies with other organisms were ruled out by using NCBI blastn program. The possibility of dimer and hairpin loop formation of oligonucleotide were minimised by analysing the sequence in Integrated DNA technology (IDT) online Oligoanalyser software.

### Hybridization assay

1 μl of DNA was mixed with 9 μl of 10 mM sodium phosphate buffer (pH 5.0). The mixture was heated to 95°C for 5 min in a water bath to denature the DNA. After denaturation, 15 μl of AuNP-oligonucleotide probe was added for hybridization. Hybridization reaction was carried out in water bath at 60°C for 15 min. Following hybridization, 0.1 M HCl was added. The colour change of AuNP-probe mixture due to aggregation was observed visually and by spectrophotometry.

### Agarose gel electrophoresis

The visual detection of the hybridized DNA was optimized by using 24-mers oligo-nucleotide complementary to probe sequences. The probe was allowed to hybridize with complementary sequences as described. These reaction mixtures were analysed on 2% agarose gel (100V for 1 h)–S1 Fig.

### Preparation of biological spiked samples

Institute animal ethics committee (IAEC) approval was obtained for all experiments and procedures conducted on animals. Bovine biological samples used in this study were semen, milk and urine. Bovine semen samples were obtained from the Germplasm centre (GPC) of the Indian Veterinary Research Institute (IVRI). Semen from pedigree bulls were collected and processed for distribution as frozen semen straw by GPC following standard guidelines, as approved by the IAEC. Bovine milk was purchased from the Livestock Product & Management (LPM) section of IVRI which sells cattle and buffalo milk. Bovine urine samples were collected during normal urination at dairy farm maintained by the LPM section of IVRI. Animals were not subjected to any experiment in the present study. The obtained biological samples were artificially infected with *Brucella* and non-*Brucella* microorganisms. Samples were first spiked with counted number of bacteria. Urine samples (1 ml) spiked with bacteria were centrifuged at 10,000 rpm for 10 min. The cell pellet was washed in ddH_2_O and harvested again by centrifugation.

Semen samples (100 μl) were spiked with bacteria and were processed by dilution with 1 ml of sterile distilled water. Milk samples were spiked with bacteria and were processed as described previously[18] with minor modification. Briefly, 0.5 ml of spiked milk sample was mixed with 100 μl of NET buffer (50 mM NaCl, 125 mM EDTA, 50 mM Tris-HCl, pH 7.6). The mixture was incubated at 80°C for 10 min and then cooled on ice. Proteinase K (20 μg/ml) was added and incubated for 90 min at 50°C. DNA was finally extracted with phenol-chloroform-isoamyl alcohol and precipitated with isopropanol.

All experiments were performed at least three times independently and by at least two different operators.

## Results and discussion

### Characterization of conjugated Au-NPs

**Fig 2:**
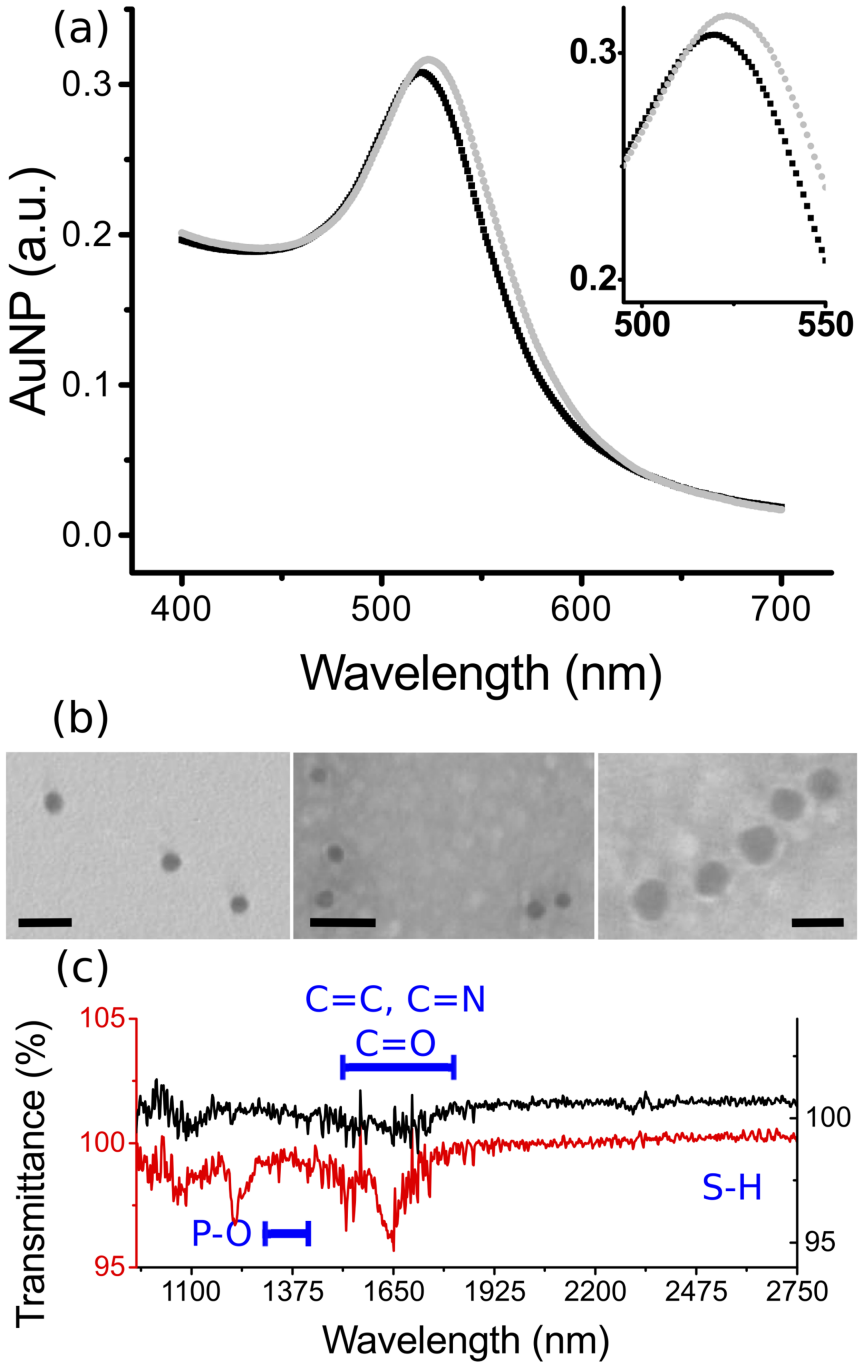
Au-NP characterization. (a) UV-Vis spectra showing a red shift of the absorbance peak from 520nm for the bare AuNP (black trace), to 524nm for AuNP conjugated with the BSCP31 probe (grey). (b) Transmission Electron Microscopy image of the colloidal AuNP before conjugation (left—scale 50 nm), AuNP conjugated with BCSP-31 probe (middle—scale 50 nm) and zoomed in (scale 20 nm). Conjugated AuNP was characterised by the presence of a halo surrounding the AuNP. (c) FTIR spectra of unconjugated (red) and conjugated (black) AuNP showing peaks at: 1000–1200 cm^-1^ that represent the vibrational spectra due to phosphate and sugar phosphate bonding; 1630–1640 cm^-1^ represent the double bond in the bases ring. Also noted is the absence of peaks at 2500–2600 cm^-1^ due to S-H vibration, indicating that the probes were conjugated onto the AuNP surface by forming Au-S bond. The full spectra are available in S2 Fig.

We characterized our newly synthesised AuNP and probe conjugated AuNP (Fig 2) using visible and IR spectroscopies, particle sizing and TEM. We first investigated their optical properties with UV-vis spectroscopy (Cary 60 UV- Vis, Agilent). The unconjugated colloidal AuNP showed a peak absorbance at 520 nm. Upon conjugation of the probe onto the AuNP, this peak shifted to 524 nm (Fig 2a), indicative of successful conjugation. This shift was similar to that obtained for a similar nanoparticle system, characterised in the same manner for exploring antibody conjugation.[19]

The probe conjugation was also validated through the measurement of the Zeta potential of the solutions. The citrate ions provide the negative charge surrounding the AuNP (potential of -9mV). The negative charge of oligonucleotides further increased the zeta potential of the particles after conjugation to -25mV. All measurements were carried out at identical pH.

The hydrodynamic diameter of our bare AuNP increased from 29.9 nm to 54.76 for AuNP-BCSP31, as measured by a Zeta sizer (Malvern Zetasizer Nano ZS). In TEM, the conjugated AuNP showed a circular hollow configuration, which was not observed in the case of bare AuNP at the same magnification (Fig 2b).

We confirmed that these changes in the AuNP is due to the binding of the DNA sequence using Fourier transform infrared spectroscopy (FTIR). The spectra shown in Fig 2c have the characteristic peaks of oligonucleotides: C = O stretch (peaks within 1665–1760 cm^-1^, and around 1630–1640 cm^-1^ in the bases ring), phosphate and sugar phosphate vibrations from the DNA backbone 1000–1200 cm^-1^). We also note the absence of the characteristic S-H vibration band (2500–2600 cm^-1^), indicating that this bond was broken to form the S-Au link during conjugation.

Hybridization was also verified using gel electrophoresis (S1 Fig). All these measurements concur to ensure the effective conjugation of our AuNP along with the validity of the assay approach.

### Assay validation

In this study, our probe-conjugated AuNP (BCSP-AuNP) was allowed to react with specific and non-specific target DNA to validate the concept of the detection and investigate the assay performance (sensitivity and specificity). Genomic DNA (gDNA) from *Brucella*, *E*.*coli* and other non-*Brucella* organisms were extracted by the NaOH digestion method. After injection of the target DNA into the AuNP solutions, the colour change upon the addition of HCl was monitored visually as well as by using absorbance. Due to the negative charges of the DNA surrounding the AuNP, the addition of HCl in the test reaction (where the complementary DNA hybridizes with the probe DNA on the AuNP) resulted in limited aggregation and the solution stayed red.

**Fig 3:**
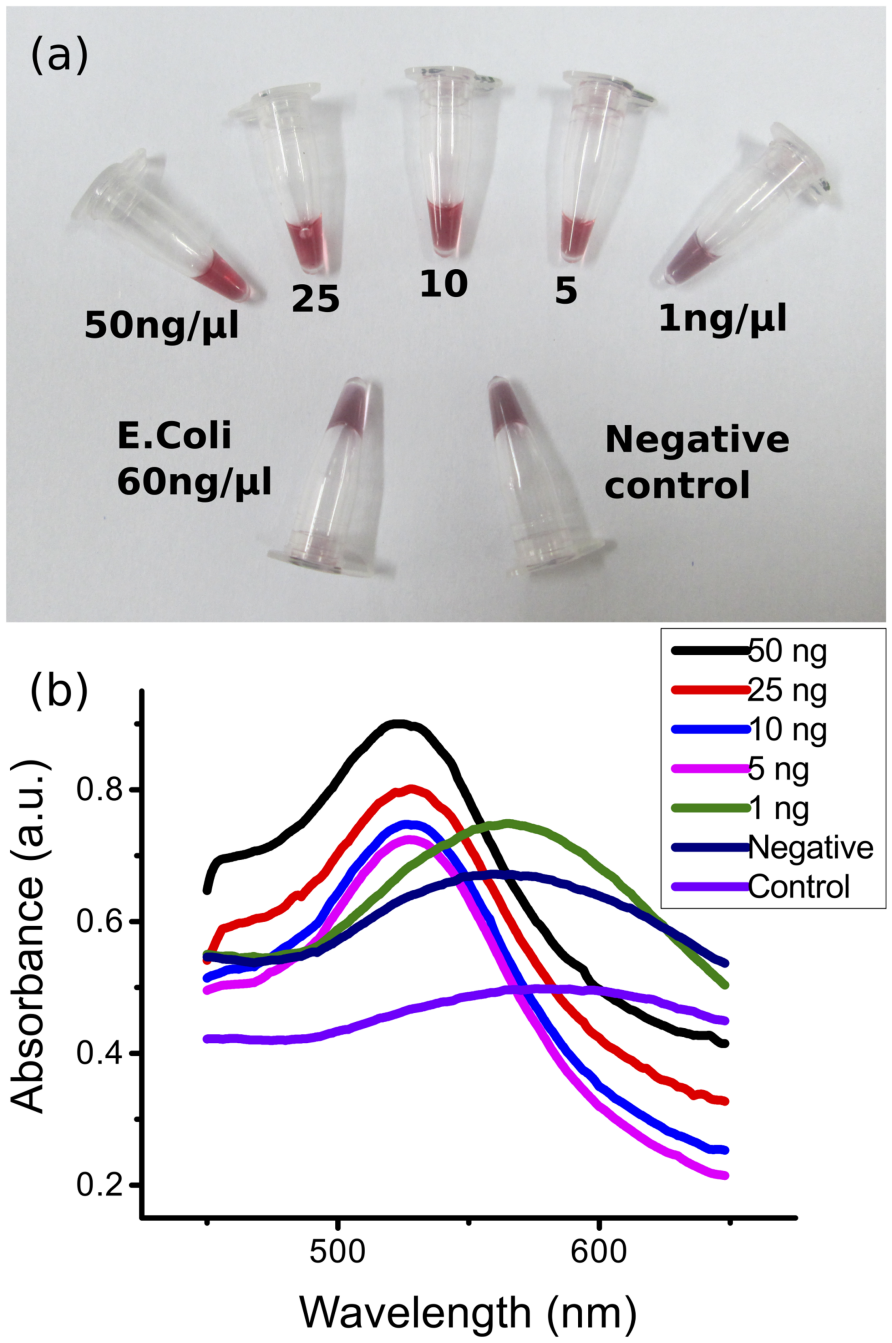
Sensitivity with genomic DNA. (a) Photographs of the assay results (after addition of HCl), for the detection of genomic DNA from *Brucella* at different concentrations (negative control is *E*.*coli* genomic DNA at 60 ng/μl) performed in tubes. Total volumes of the reaction mix in each tube is 25 μl. Quantities are indicated for 1 μl of sample before dilution in the reaction mix. (b) UV-Vis spectra of gDNA sensitivity showing the absorbance peak at ~520nm for positive samples and red shifting with absorbance peak at higher wavelength in both negative and control samples, with a limit of detection of 5ng/μl in the sample before dilution in the reaction mixture. S3 Fig. Enhanced photographs of all figures (3–6) are available as supplementary information to provide more apparent color differentiation.

In contrast, when the target DNA was non-complementary (e.g. *E*.*coli* DNA), the AuNP aggregated readily, giving the color purple to the reaction (Fig 3a). This direct visual observation was confirmed spectroscopically, showing that positive samples exhibited the characteristic absorbance peak at around 520 nm. The negative controls generated a spectral shift with peaks above 560nm (Fig 3b).

### Sensitivity and specificity of AuNP-oligonucleotide probes

**Fig 4:**
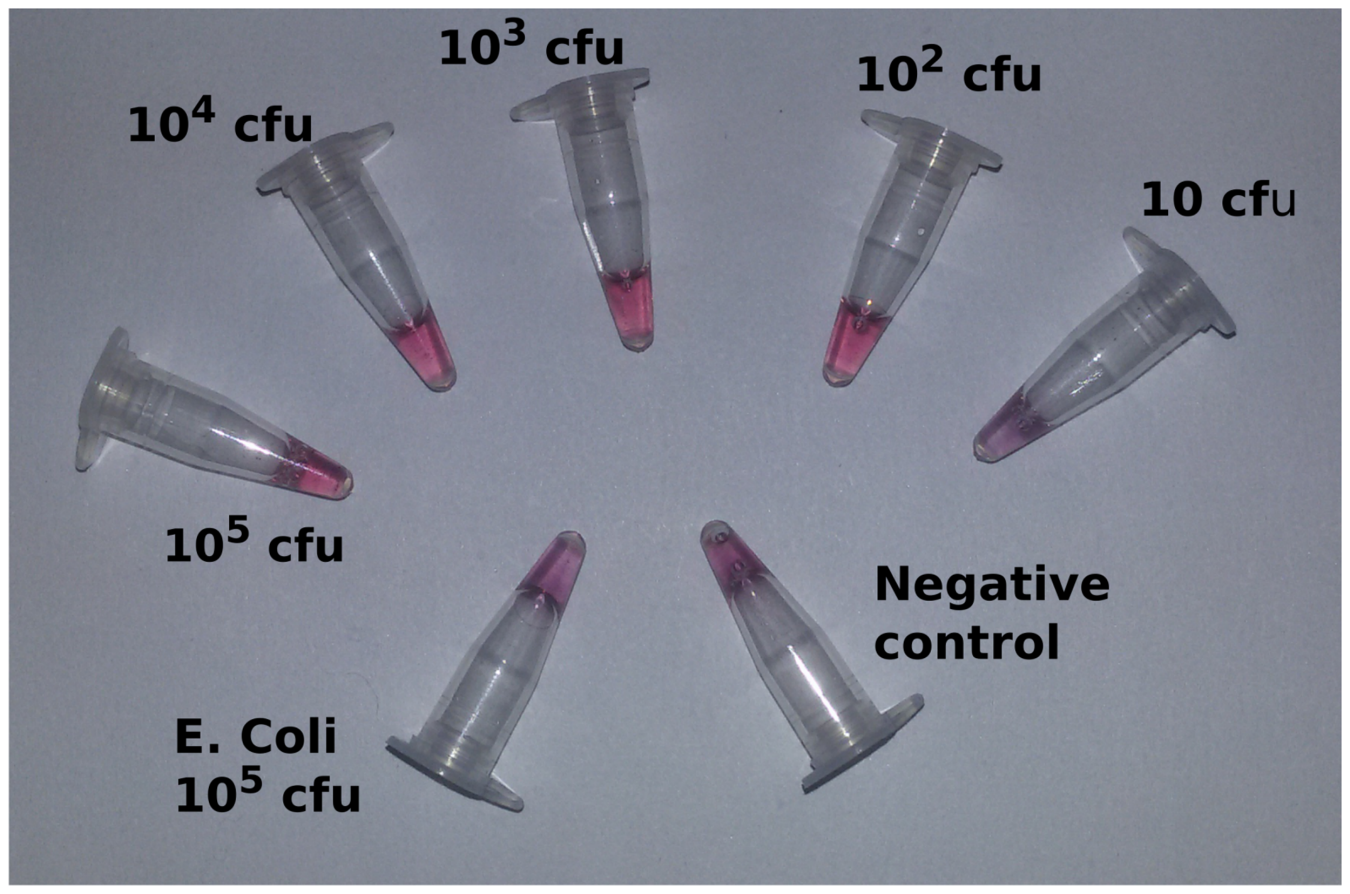
Sensitivity with bacteria cultures for BCSP31-AuNPs. Quantities indicated are in one ml (25 μl total volume of reaction). The assay could detect up to 10^2^ cfu/ml. Enhanced in S4 Fig.

Fig 3a shows the visual sensitivity of the assay. *Brucella* gDNA at different concentration was used for hybridization assay. *E*.*coli* DNA was used as negative control. Addition of HCl resulted in a change of colour from red to purple in the negative control (unhybridised), whereas in the positive reactions (hybridized, e.g. at 50ng/μl), the colour of AuNP-probe remained red. The sensitivity of the BCSP31 probe was found to be 5ng/μl. When challenged with DNA isolated from pure bacteria cultures, the detection limit was 10^2^ cfu/ml (Fig 4).

**Fig 5:**
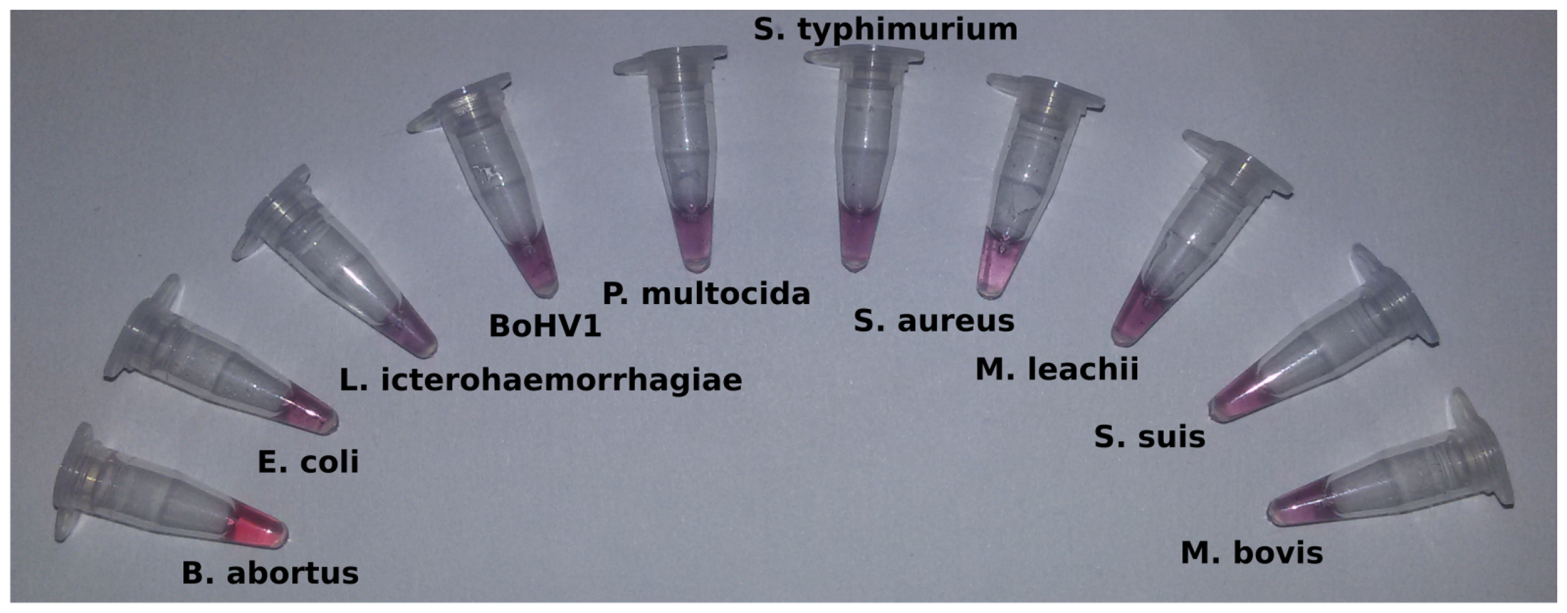
Specificity. Reactions for *Brucella spp* and nine non-*Brucella spp* (*E*.*coli*, *L*. *icterohaemorrhagiae*, *BoHV1*, *P*. *multocida*, *S*. *typhimurium*, *S*. *aureus*, *M*. *leachii*, *S*. *suis* and *M*. *bovis*) with BCSP-AuNP. Changes in colour were observed in all DNAs samples except in *Brucella* DNA (tube on the left).

To determine the specificity of the assay, non-*Brucella* microorganisms (*E*.*coli*, *L*. *Icterohaemorrhagiae*, *P*. *multocida*, *S*. *typhimurium*, *S*. *aureus*, *M*. *leachii*, *S*. *suis and M*. *bovis*) and a virus (BoHV1) were subjected to hybridization with AuNP-probes after DNA extraction. All non-*Brucella* samples showed a colour change to purple (Fig 5). This indicates a high specificity for the designed probe towards the *Brucella spp*.

*Brucella* infected animals routinely shed organisms in the natural secretion and excretion liquids such as milk, urine and uterine discharges. The detection of *Brucella* in semen has a specific interest as a consequence of recent changes in animal husbandry driven by the need to improve agricultural efficiencies to feed a rapidly expanding population, particularly in India. For example, in order to achieve a step change in the production of milk, artificial insemination of cattle is now common. In such facilities, it is widely known that *Brucella* infections amongst the donor can lead to reduced fertility and in some circumstances spontaneous abortion.

**Fig 6:**
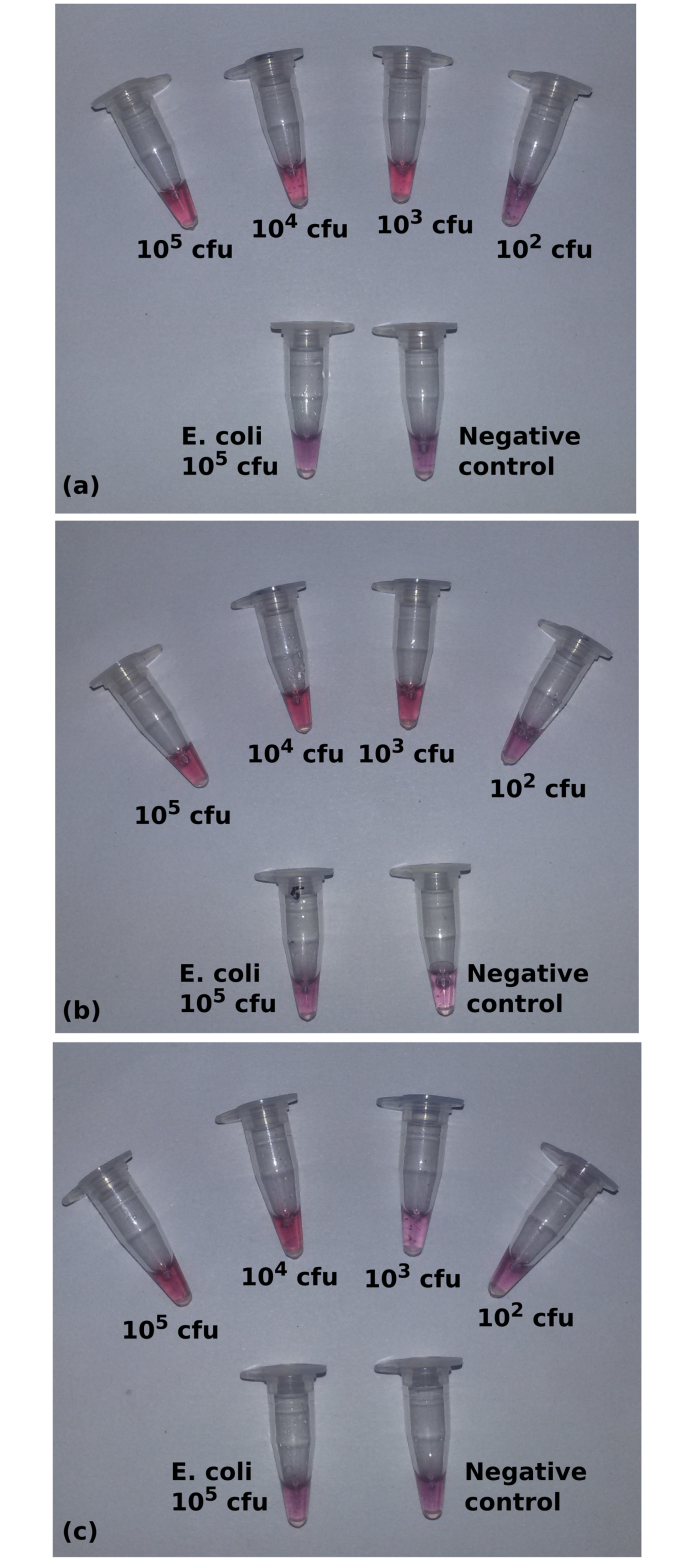
Sensitivity of the assay in spiked samples of (a) urine (b) semen and (c) milk. The mechanism of spiking is described in detail in the text. Enhanced in S5 Fig.

To study the feasibility of detection of *Brucella* using the designed AuNP-probes, we spiked bovine semen, milk and urine samples with bacterial count ranging from 10^5^ cfu/ml to 10^2^ cfu/ml. Artificially spiked biological samples were processed for DNA extraction and the hybridization assay was performed. The assay could detect organism copy numbers down to 10^3^ cfu/ml of *Brucella* organisms in semen and urine samples. In the case of milk, the detection limit was 10^4^ cfu/ml (Fig 6). This lower performance could be due to the presence of fat in which some organisms may get trapped.[20] This sensitivity is on par with the bacterial loading commonly seen in contaminated samples, which ranges from 100 to 50,000 organisms/ml [21].

## Conclusions

Current techniques for the determination of *Brucella* are not wholly appropriate for the needs of rural counties, particularly in LMICs, where there may be reduced access to infrastructure. In this study, we have demonstrated a simple and sensitive AuNP based detection of *Brucella spp*. with high specificity. Our test relies upon the plasmonic colour change when specific DNA hybridization events occur at a metal nanoparticle, thus needing only visual inspection of the results. The hybridization reaction does not require more than a heater (no thermal cycling, no extensive control required) or technical expertise beyond the addition of the sample. In terms of cost and convenience, the assay can be implemented readily in the field. We show that this assay can be used for detection of bacteria from milk, semen and urine which are considered the most relevant samples for the diagnosis of reproductive diseases, including brucellosis.

This assay could be performed within 30 min. The visual detection limit of 10^3^ cfu/ml *Brucella* organism in biological samples is appropriate for the translation of the assay at the “point-of-care”. Importantly, in the future, this test could either be used for selection of bulls for semen collection at germplasm centres, for the examination of frozen semen before artificial insemination or for the examination of bulk milk before packaging, to ensure the safety of consumers.

## Supporting information

S1 Fig Agarose gel-electrophoresis. (2% agarose W/V) of AuNP conjugated with DNA sequence: Lane 1: only BCSP31-AuNP; Lane 2: BCSP31-AuNP with hybridized with non-complementary sequence; Lane 3: BCSP31-AuNP hybridised with target sequence. Upon hybridisation, a retardation is observed.
(TIFF)

S2 Fig Combined full FTIR spectra. (TIFF)

S3 Fig Fig 4 with contrast enhanced. (TIF)

S4 Fig Fig 5 with contrast enhanced. (TIF)

S5 Fig Fig 6 with contrast enhanced. (TIF)
