# Supplementary figures and images for: Visual detection of Brucella in bovine biological samples using DNA-activated gold nanoparticles
Source: PLoS One. 2017 Jul 18;12(7):e0180919. doi: 10.1371/journal.pone.0180919 (PMC5515423; doi:10.1371/journal.pone.0180919)

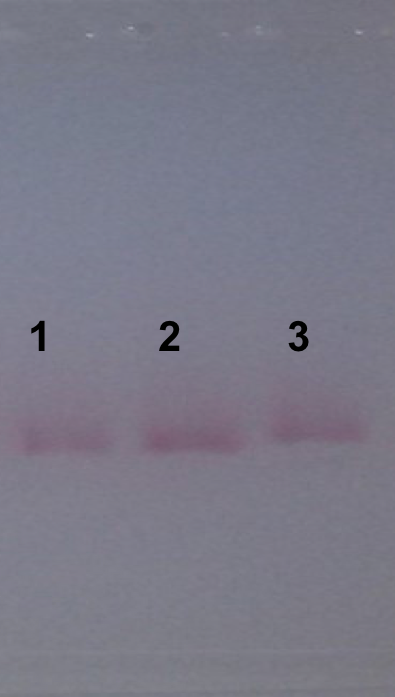

Supplement: S1 Fig — (2% agarose W/V) of AuNP conjugated with DNA sequence: Lane 1: only BCSP31-AuNP; Lane 2: BCSP31-AuNP with hybridized with non-complementary sequence; Lane 3: BCSP31-AuNP hybridised with target sequence. Upon hybridisation, a retardation is observed. (TIFF) [file pone.0180919.s001.tiff]

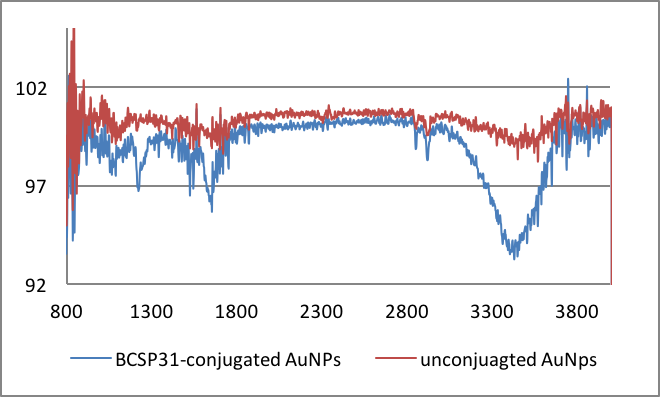

Supplement: S2 Fig — (TIFF) [file pone.0180919.s002.tiff]

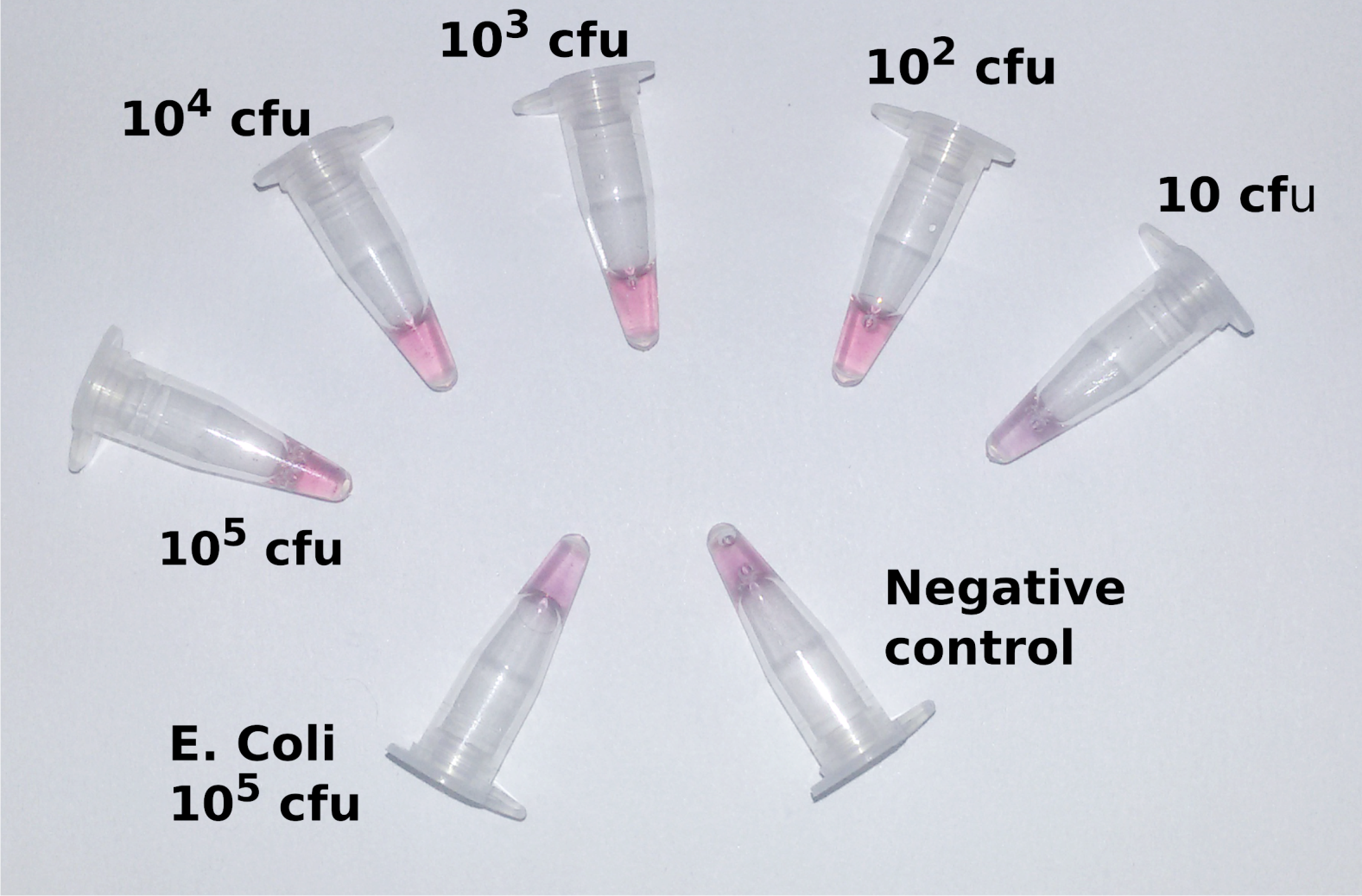

Supplement: S3 Fig — (TIF) [file pone.0180919.s003.TIF]

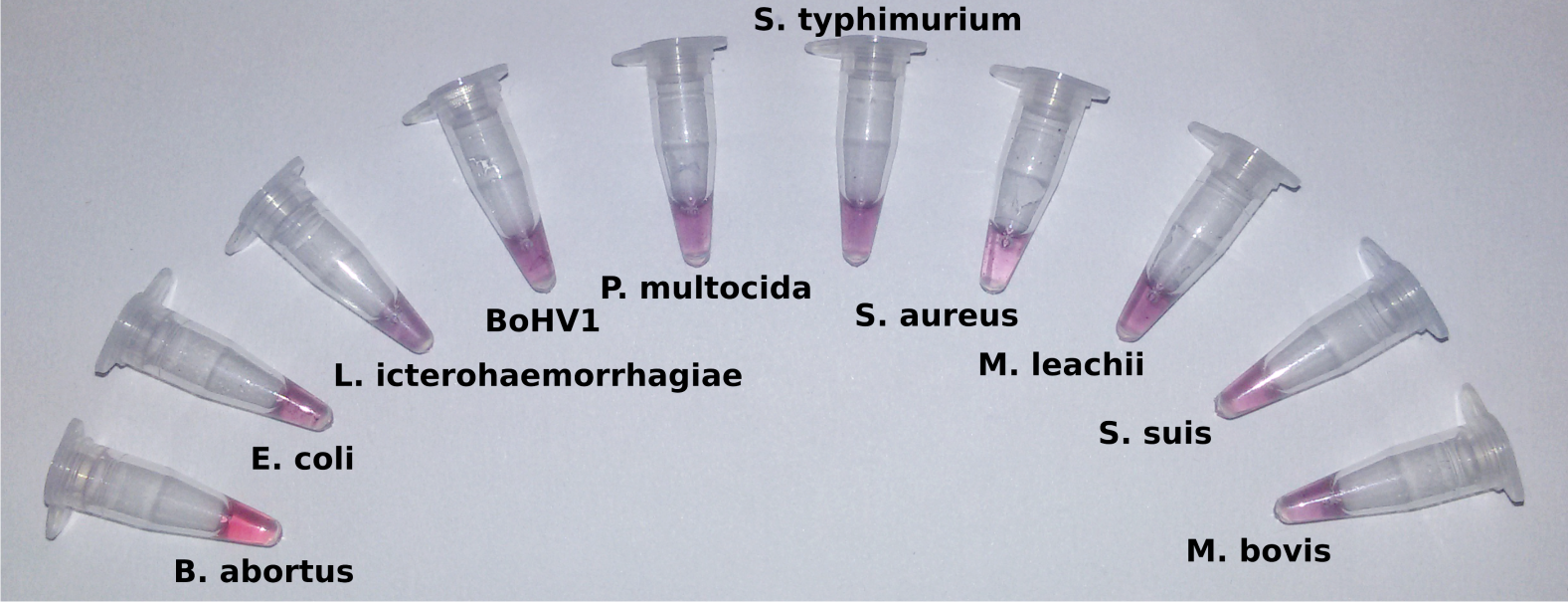

Supplement: S4 Fig — (TIF) [file pone.0180919.s004.TIF]

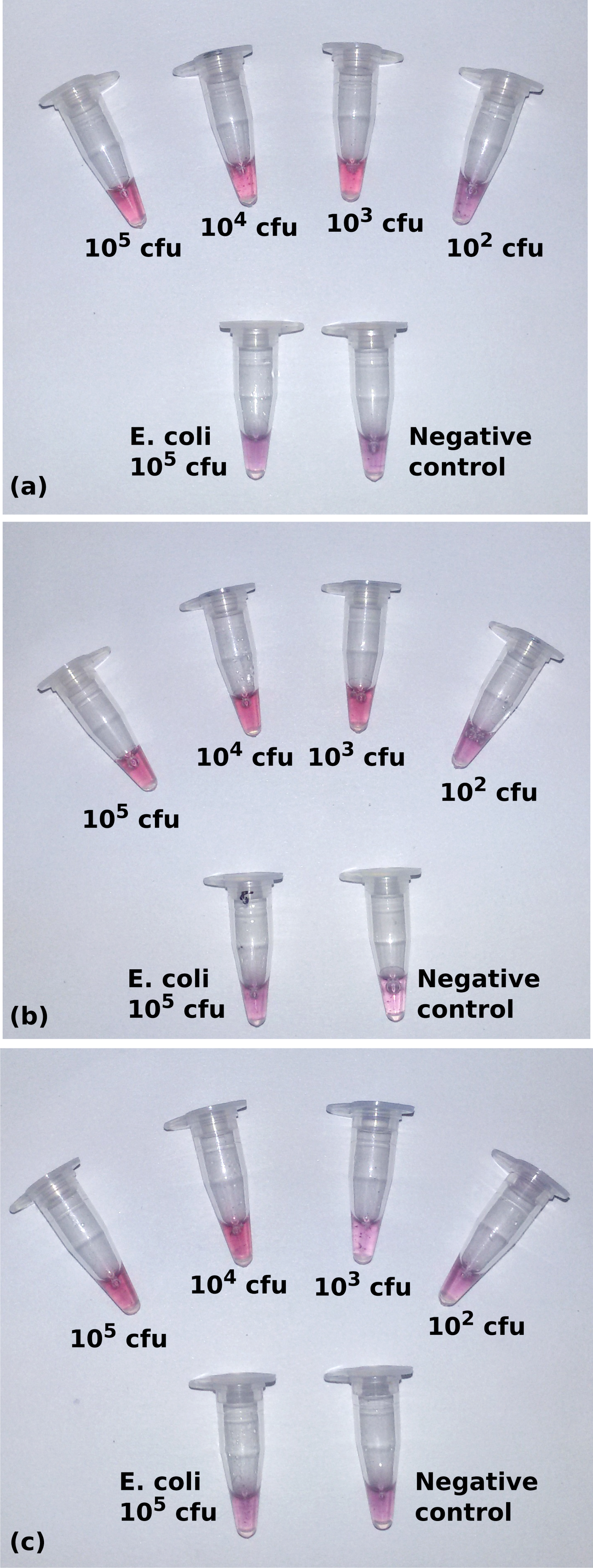

Supplement: S5 Fig — (TIF) [file pone.0180919.s005.TIF]
